# Improving infection control in a veterinary hospital: a detailed study on patterns of faecal contamination to inform changes in practice

**DOI:** 10.1186/s13620-023-00229-w

**Published:** 2023-02-13

**Authors:** Ashokkumar Singaravelu, Bernadette Leggett, Finola C. Leonard

**Affiliations:** 1 UCD School of Medicine, University College Dublin, Dublin 4, Ireland; 2 UCD School of Veterinary Medicine, University College Dublin, Dublin 4, Ireland

**Keywords:** Infection control, Veterinary hospital, Faecal organisms, Contamination, Nosocomial infection

## Abstract

**Background:**

The main purpose of this study was to investigate the cleanliness and microbial burden of a veterinary hospital to establish the extent of cross-contamination with faecal bacteria as an aid to reducing nosocomial infections. Enterococci and *Escherichia coli* were used as faecal indicator organisms as they can survive on inanimate surfaces for months and pose a threat to animal health.

The study consisted of several elements: (i) a cross-sectional study to identify sites currently contaminated with faecal organisms that could be usefully included in a longitudinal study, (ii) a 3-week longitudinal study to identify sites from which faecal bacteria were repeatedly recovered, (iii) once-off monitoring of hand hygiene, (iv) a review of all hospitalised cases with confirmed *E. coli* or enterococcal infection during the 8-week study period to investigate possible hospital-acquired (HAI) infection and relationship with environmental contamination. Environmental surface and hand hygiene were assessed using 3M™ Clean-Trace™ ATP test, 3M™ Petrifilm™ plates and bacteriological culture of *Enterococcus* species and *E. coli*. Cross contamination was assessed using results of antimicrobial susceptibility testing.

**Results:**

In the cross-sectional study, 26 of 113 (24.5%) of sites sampled exceeded the accepted microbial threshold (2.5 CFU/cm^2^) and *Enterococcus* species were isolated from 31 (27.4%) and *E. coli* from 9 (7.9%) of 113 samples. Organic residue and microbial levels were high in the dog kennels even after cleaning and faecal organisms were also recovered from sites such as the dispensary, a student computer and staff common room. Four of 51 (7.8%) hand samples were contaminated with faecal bacteria. Nine sites were monitored on three occasions in the longitudinal study and a total of 23 *Escherichia coli* and 6 *Enterococcus* species were recovered. Seven of the nine sites were positive for faecal organisms on more than one occasion. There was no change in cleanliness or microbial burden over 3 weeks. Twenty-one of the 73 isolates (28.8%) recovered during all parts of the study were multi-drug resistant. Enterococci and *E. coli* isolates with similar resistance patterns were recovered from the environment in the large and small animal hospitals and from a small number of patients during the same timeframe, suggesting possible hospital acquired infections.

**Conclusions:**

Results suggested that movement between the small and large animal hospital areas may have been responsible for cross-contamination and possible hospital-acquired infections. The data show that cross-sectional and longitudinal monitoring of faecal contamination across all hospital areas can play an important role in informing review of infection control protocols in veterinary hospital settings. Changes in practices in the hospital based on results generated are outlined.

**Supplementary Information:**

The online version contains supplementary material available at 10.1186/s13620-023-00229-w.

## Introduction

The practice of veterinary medicine has changed in recent decades with an increase in the number of specialised secondary and tertiary referral practices. There are increasing numbers of patients, especially in companion animal practice, that are treated for chronic illnesses, immunosuppressive conditions or have received surgical implants. In addition, many animals presenting at veterinary referral hospitals have previously received treatment with antimicrobial drugs. In primary practice, Singleton et al. reported prescription of antimicrobials in 18.8% of dogs and 17.5% of cats presenting to 457 sentinel practices in the UK [1]. The figures for referral practices are likely to be much higher and Edmondson et al. (2022) state that almost 50% of animals presenting to a small animal referral hospital had received antimicrobials [2]. As in human medicine, such patients are at particular risk of acquiring infection with multi-drug resistant organisms and effective infection control procedures are essential to minimise this risk [3]. There are major differences between animal and human hospitals including higher levels of faecal material, hair and dust. Equine, farm and companion animal facilities may be located on the same site with movement of staff and, in university veterinary hospitals, students, between different areas.

The University College Dublin Veterinary Hospital (UCDVH) has a comprehensive infection control policy and routine surveillance of environmental hygiene throughout the hospital is conducted at least once a year. In addition, further monitoring of kennels and treatment areas is undertaken following discharge of animals diagnosed with high-risk infections such as methicillin-resistant *Staphylococcus aureus* (MRSA), methicillin-resistant *Staphylococcus pseudintermedius* (MRSP) or other multidrug resistant (MDR) or zoonotic pathogens. Surveillance is conducted using Adenosine Triphosphate (ATP) bioluminescence measurement and conventional bacteriological culture methods. Routine monitoring is carried out using ATP measurement only, with conventional bacteriology conducted if deemed necessary following assessment of the risk by the infection control team. ATP bioluminescence tests measure organic residue levels and have the major advantage that they give a virtually instant result compared to traditional culture methods that require 18–36 h or longer. Such tests can therefore be a huge help to infection control staff members when they are required to make a rapid decision whether a room or equipment can be used safely.

Monitoring and surveillance results in UCDVH show that although results are usually within acceptable limits, there are some areas within the hospital where faecal organisms are frequently recovered despite the hygiene measures employed. In addition, occasional identification by laboratory staff of apparent clusters of infection with organisms with the same antimicrobial resistance pattern in two or three patients within a short timeframe suggests the occurrence of hospital acquired infections. Faecal bacteria such as enterococci and *Escherichia coli* can survive on dry inanimate surfaces for months [4, 5]. These pathogens have been identified on hand-touch surfaces and equipment in veterinary hospitals which may contribute to nosocomial infections [6–8].

The overall objective of this study was to identify the main areas within the veterinary hospital from which faecal organisms could be repeatedly recovered with the aim of using results to re-evaluate the cleaning schedules for these areas, including consideration of more frequent and /or changes to cleaning methods. It was also suspected that cross-contamination between areas occurred at least occasionally, including between the large and small animal hospitals. A second objective was to presumptively identify such cross-contamination using antimicrobial resistance typing and evaluate its significance.

## Methods

### Design of the study

This study was conducted at University College Dublin Veterinary Hospital (UCDVH) from 25th May to 9th July 2021 and consisted of several elements: (i) a cross-sectional study to identify sites currently contaminated with faecal organisms that could be usefully included in a longitudinal study, (ii) a 3-week longitudinal study to identify sites from which faecal bacteria were repeatedly recovered, (iii) once-off monitoring of hand hygiene and (iv) a review of all hospitalised cases with confirmed *E. coli* or enterococcal infection during the 8-week study period to investigate possible hospital-acquired (HAI) infection and the relationship with environmental contamination.

#### Cross-sectional study

A cross-sectional study was done to identify sites currently contaminated with antimicrobial-resistant *enterococci* and *E. coli* to evaluate the extent of contamination and to identify possible sites for inclusion in a longitudinal study*.* A total of 113 samples were collected including surfaces such as floors, drains, worktops, and high touch surfaces (Table 1). High touch non-critical environmental surfaces included doors, kennel bars and bolts, computer keyboards, taps and re-usable patient care equipment such as stands for intravenous fluid administration [9].

**Table 1:** List of surface types sampled in a cross-sectional study of a university veterinary hospital

Surface	Sampling area
Floors	36 cm^2^
	36 cm^2^
Worktops	36 cm^2^
Keyboards & mouse	140 cm^2^
Handles	44 cm^2^
Telephones	20 cm^2^
Door handles	44 cm^2^
Taps	20 cm^2^
Fluid pumps	80 cm^2^
Couches	36 cm^2^
Pens	11 cm^2^
Anaesthetic machines	40 cm^2^
Bair Hugger warming devices	40 cm^2^
Shoreline concrete of kennels	36 cm^2^
Syringe drivers	45 cm^2^
Kennel door handles	25 cm^2^
X-ray machine	40 cm^2^
Ultrasound machine	40 cm^2^
Fridge/freezer door handles	30 cm^2^

#### Longitudinal study

A prospective longitudinal study was performed to identify sites acting as reservoirs of faecal bacteria and possible sources of nosocomial infection. Nine areas were selected for inclusion from the cross-sectional study based on recovery of faecal organisms, results of antimicrobial resistance typing and type of animal clinics (small or large animal clinics). Samples were collected on Monday (11 AM – 12 PM) every week for 3 weeks (Table 2).

**Table 2:** List of sites selected for the longitudinal study to identify reservoir sites of faecal contaminants

	Site
1	Large animal treatment room (Keyboard & mouse)
2	Large animal treatment room (Floor)
3	Small animal treatment room (Keyboard & mouse)
4	Small animal treatment room (Floor)
5	Small animal treatment room (Syringe driver)
6	Intensive Care Unit (Floor)
7	Dog medicine ward (Floor)
8	Dog surgery ward (Floor)
9	Corridor connecting large animal & small animal sites (Floor)

#### Hand sampling

Hand sampling was conducted during the longitudinal study to evaluate whether the same faecal organisms isolated from the environment were also contaminating hands. According to COVID-19 protocols, the hands of veterinary professionals and students were self-sampled using pre-moistened cotton-tip swabs. Participants swabbed the entire palmar surface of their dominant hand. Samples were collected anonymously and oral consent was obtained; 51 hand samples were obtained on five occasions based on willingness of personnel to participate when approached (the person collecting samples was not known to hospital personnel). The same individual was not sampled on more than one occasion.

#### Retrospective review of patient records

All hospitalised cases with confirmed *E. coli* or enterococcal infection during the 8-weeks study period were reviewed to investigate possible hospital-acquired (HAI) infection and relationship with environmental contamination. The resistance profile of isolates from hospitalised cases was analysed by VITEK®2 AST Card and VITEK® 2 Systems Version: 08.02 (bioMérieux, France). The VITEK®2 cards used for testing enterococcal isolates do not include vancomycin analysis. The VITEK®2 GN97 cards for *E. coli* isolates analyse resistance to 19 antimicrobials and test for presumptive extended-spectrum beta-lactamase (ESBL) production. The cards used for *Enterococcus* species analyse resistance to 19 antimicrobials. The resistance profiles of suspected HAI cases were compared to those of faecal bacteria isolated from the environment. In our study, we used 6 antimicrobials for *E. coli* and 4 for *Enterococcus* species in the disc diffusion test (resources for the project did not allow testing using the VITEK® 2 System).

### Hygiene evaluation and microbiological methods

#### *ATP *bioluminescence* test *

ATP bioluminescence tests measure the organic residue present on the surface. 3M™ Clean-Trace™ Surface ATP Test Swabs UXL100 were used according to the manufacturer’s instructions. Depending on the type of surface, the area of the sampling surface varied (Table 1). All ATP swabs were analysed using 3M™ Clean-Trace™ NGi Luminometer immediately after swabbing. A cut-off value of 500 relative light units (RLU) is deemed acceptable in the UCDVH based on recommendations of the supplier and pilot in-house data generated following purchase of the device.

#### Use of Petrifilm TM plates & microbiological culture

For each site sampled, two adjacent areas were sampled, one with a Letheen broth swab sampler (10 ml) (3M™) and one with an ATP swab. One ml of the test sample was inoculated onto an area of approximately 20 cm^2^ of an aerobic count Petrifilm (3M™) plate. The Petrifilm plates were incubated at 30 °C for 24 h and the number of aerobic bacterial colonies was counted manually. Plates were incubated for a further 24 h if growth was sparse or colonies were barely visible. If the total number of colonies was too large to count, all colonies present in 1 cm^2^ of the plate were counted and the number multiplied by 20 to obtain a total count per 1 ml of inoculated broth. A cut-off value of < 2.5 colony forming units (CFU)/cm^2^ was used, similar to that used in human hospitals [10]. The remainder of the Letheen broth samples was incubated at 37 °C for 18 h followed by subculture onto MacConkey Agar No.2 plates for isolation of *E. coli* and enterococci. Plates were incubated for 18 h and up to 36 h if there was no growth after 18 h. One isolate of presumptive *E. coli* and *Enterococcus* species per sample was selected for further investigation in the cross-sectional study. For the longitudinal study, up to 5 isolates of each organism were selected for identification and antimicrobial resistance typing. As environmental samples are likely to contain isolates from different sources with diverse phenotypes, this methodology was selected to increase the likelihood of identification of cross contamination between sites. Gibbons et al. also tested up to five isolates from pooled samples to ensure representative results of *E. coli* resistance patterns [11].

Isolates of presumptive *E. coli* were identified using indole and citrate tests and presumptive enterococcal isolates that were of interest based on the similarity of their antimicrobial resistance patterns, were speciated using VITEK®2 GP ID Card and VITEK® 2 Systems Version: 08.02 (bioMérieux, France).

Hand sample swabs were inoculated onto MacConkey Agar No.2 plates and incubated at 37 °C for 18 to 36 h. Isolates of presumptive *E. coli* and enterococci were identified as for environmental samples.

#### Antimicrobial susceptibility tests

The susceptibility of *E. coli* and enterococci isolated from environmental samples was analysed using the Kirby Bauer method and results were interpreted as either resistant or susceptible using CLSI 2018 Vet 08, CLSI 2018 Vet01, and EUCAST 2021 guidelines if CLSI breakpoints were not available for a particular agent [12–14]. *Enterococcus* species were tested for susceptibility to enrofloxacin (ENR) 5 μg, vancomycin (VA) 30 μg, tetracycline (TE) 30 μg and amoxycillin-clavulanic acid (AMC) 30 μg on Mueller–Hinton agar supplemented with 5% defibrinated sheep’s blood. Cephalothin (KF) 30 μg, ENR 5 μg, TE 30 μg, AMC 30 μg, trimethoprim-sulphamethoxazole (SXT) 25 μg, and cefpodoxime (CPD) 10 μg discs were used for *E. coli* testing. Detection of AmpC- and extended-spectrum β-lactamase (ESBL)-positive *E. coli* was confirmed according to EUCAST guidelines [15]. For the longitudinal study, up to five isolates from each sample were selected from MacConkey Agar No.2 plate for susceptibility testing. Multidrug resistant (MDR) bacteria were defined as resistant to at least one agent in three or more antimicrobial classes [16].

### Statistical analysis

The Mann–Whitney test was used to compare ATP readings and microbial burden of large and small animal areas. The Kruskal–Wallis test was used to compare RLU/cm^2^ and CFU/cm^2^ values with respect to surface and site. If the result was significant, posthoc analysis (Dunn test, method: holm) was performed to determine which surfaces/sites differed significantly from each other. As only week 3 RLU data was not normal, analysis of variance with repeated measures testing was used to compare the RLU readings of 9 sites over the 3-week period of the longitudinal study. The CFU/cm^2^ data of the longitudinal study was not normally distributed and hence, a Friedmann test was used for analysis. Correlation between RLU/cm^2^ and CFU/cm^2^ was examined using Spearman’s rank correlation test. The effect size for all tests was calculated. All analyses were conducted in RStudio Version 1.4.1717 (R version 4.1.0 (2021–05-18)) [17]. All results were deemed significant at α = 0.05.

## Results

### Cross sectional study

Twenty-six of 113 (24.5%) of sites sampled exceeded the accepted microbial threshold (2.5 CFU/cm^2^) (Table 3 and Figures S1,S2,S3,S4). Median ATP readings for the different sites are also shown in Table 3 and Figures S1,S2,S3,S4. Readings differed between the following surfaces: (1) Floor and high touch surfaces (*p* < 0.001), (2) Door and kennel (*p* = 0.01), (3) Kennel and high touch surface (*p* < 0.001) and (4) Kennel and other surfaces (*p* = 0.027). A moderate positive correlation was found between ATP readings and aerobic colony counts (*p* < 0.0001, effect size = 0.4076).

**Table 3:** Summary results of hygiene evaluation data from a cross-sectional study of 113 sites in a University Veterinary Hospital, according to A, type of surface and B, site sampled

Characteristics (No. of samples)	Median RLU/cm^2^ (interquartile range)	Median CFU/cm^2^ (interquartile range)	Number (%) of samples exceeding the microbial threshold (≤ 2.5 CFU/cm^2^)	Number (%) of sites with faecal bacteria isolated	
				***Enterococcus* species**	***Escherichia coli***
A. Type of surface					
Floor (18)	199.6 (128.2 – 478.7)	0.7 (0.1 – 5.8)	6/18 (33.3)	4/18 (22.2)	3/18 (16.6)
Door, fridge door, and freezer door (21)	100.6 (40.8 – 307.9)	0.3 (0.2 – 2.0)	2/21 (9.5)	3/21 (14.3)	0/21
Tap and worktop (11)	146.4 (75.9 – 201.9)	0.5 (0.1 – 2.3)	3/11 (27.3)	0/11	0/22
Dog kennel: floor, drain, door handle, and shoreline concrete (10)	575.9 (283.8 – 1001.6)	3.1 (0.2 – 18.1)	5/10 (50.0)	5/10 (50.0)	2/10 (20.0)
High touch surfaces (HTS): Keyboard & mouse, fluid pump, syringe driver, anaesthetic machine, Bair Hugger warming device, portable SpO_2_, X-ray machine, ultrasound machine, and telephone (45)	47.6 (22.6 – 124.8)	0.5 (0.1 – 1.3)	10/45 (22.2)	16/45 (35.5)	4/45 (8.9)
Other: couch, bed, instrument trolley, table, pen, and handle (8)	92.7 (25.6 – 172.3)	0.8 (0 – 1.4)	0/8	3/8 (37.5)	0
All surfaces (113)			26/113 (24.5)	31/ 113 (27.4)	9/113 (7.9)
B. Site sampled					
B1. Small animal					
Consult room, induction room, ICU, medicine treatment room, dog kennels, and surgery room (58)	132.9 (35.8 – 446.2)	0.6 (0 – 1.4)	12/58 (25)	21/58 (36.2)	5/58 (8.6)
Emergency clinic (14)	64.8 (24.7 – 140.7)	0.3 (0 – 0.9)	2/14 (14.3)	4/14 (28.6)	0/14
Operating theatre for 'dirty' procedures(4)	67.8 (41.4 – 94.7)	0.1 (0 – 0.2)	0/4	1/4 (25)	0/4
B2. Large animal					
Treatment room, surgery suite, and tutorial room (18)	170.6 (88 – 278.4)	2.2 (0.6 – 18.1)	8/18 (44.4)	3/18 (16.7)	3/18 (16.7)
B3. Area shared by small and large animal clinics					
Diagnostic imaging room (X-ray & ultrasound machines) (5)	46.8 (9.9 – 77.6)	0.3 (0.1 – 0.5)	0/5	0/5	0/5
B4. Other locations					
Staff common room and nurses’ tea station (5)	131.8 (74.9 – 146.4)	0.3 (0 – 1.6)	1/5 (20.0)	0/5	0/5
Corridor, outside small animal ward, reception, student computer, and dispensary (9)	105.5 (67.2 – 165.3)	0.5 (0.2 – 3.1)	3/9 (33.3)	2/9 (22.2)	1/9 (11.1)
All sites (113)			26/113 (23)	31/113 (27.4)	9/113 (7.9)

*Enterococcus* species were isolated from 31 (27.4%) and *E. coli* from 9 (7.9%) of 113 samples. *Enterococcus* spp. were found in the dog kennels and on high touch surfaces, and in terms of sites, more isolates were recovered from the small animal than from the large animal area.

Organic residue and microbial levels were high in the dog kennels; enterococci and *E. coli* were identified on 5/10 and 2/10 surfaces associated with this area respectively. Enterococci were identified in samples collected at the drains, on the floors and at the shoreline concrete of the kennels. *E. coli* were identified on the floors. At the time of sampling, the kennels were vacant, had been cleaned and were ready for use.

In terms of site, the large animal areas had numerically higher ATP readings (*p* = 0.411) and statistically higher (*p* = 0.003) microbial burden compared to the small animal areas. The proportion of faecal contaminants identified in the small animal sites (21/58; 36.2%) was numerically higher than in the large animal sites (3/18; 16.7%).

Among the surfaces sampled using Petrifilm plates, the most contaminated surfaces exceeding the threshold included the floors (9/22), the dog kennels (2/5), and high touch surfaces (10/45).

Faecal bacteria were recovered from sites such as the dispensary, outside the small animal ward, and a student computer. Faecal organisms were recovered from areas occupied by staff such as the nurse's tea station and staff common room.

Although ATP readings were low on high touch surfaces, 10/45 (22%) exceeded the microbial threshold (< 2.5 CFU/cm^2^) and 20/45 (44.4%) of surfaces were contaminated with faecal organisms. We recovered 8 (50.0%) *Enterococcus* spp. and 2 (12.5%) *E. coli* isolates from 16 samples of keyboards and mouses. Two of 7 fluid pumps were contaminated with *Enterococcus* spp. and 1/5 syringe drivers were contaminated with *E. coli*. In total 7 telephones were sampled, and 4 *Enterococcus* spp. and 1 *E. coli* were recovered from them. *Enterococcus* spp*.* were recovered from an anaesthetic machine and an ultrasound machine (Table 3). Other surfaces sampled had low levels of microbial contamination.

### Longitudinal study

**Fig. 1:**
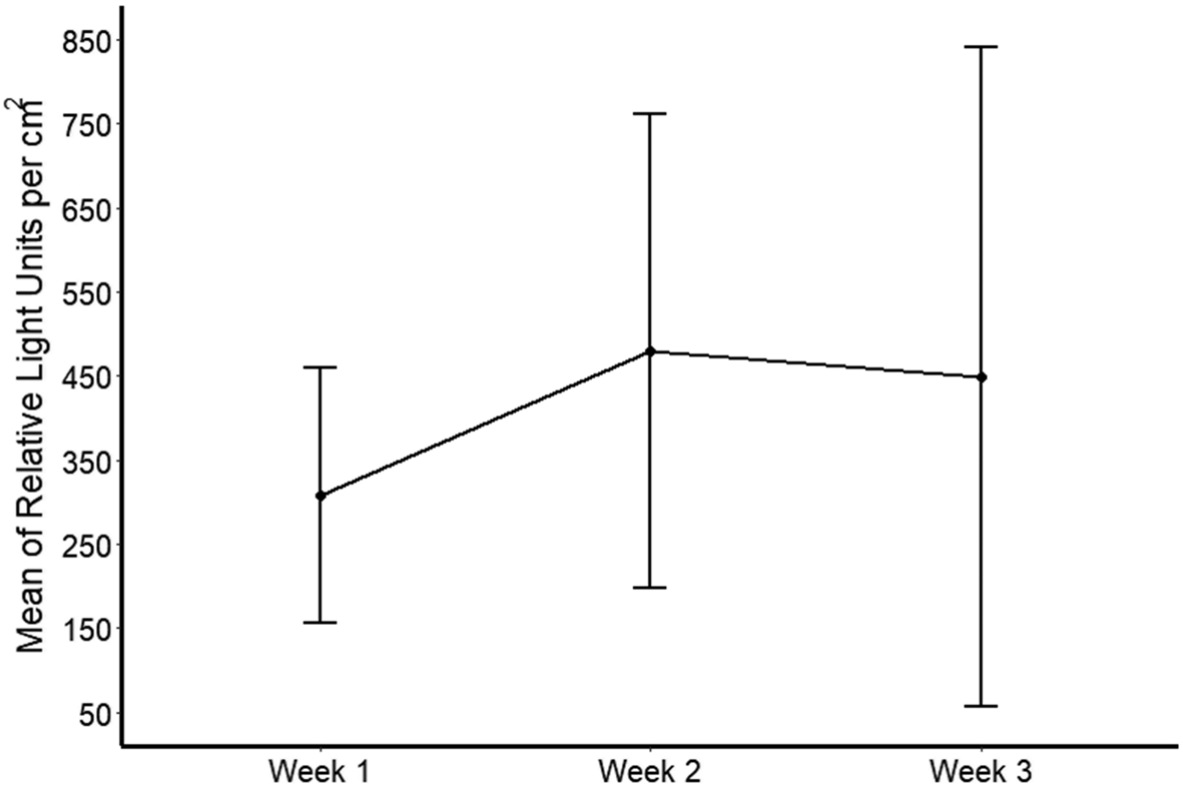
Mean and 95% confidence interval of ATP measurements in Relative Light Units per cm^2^ of nine sites sampled on three occasions

**Fig. 2:**
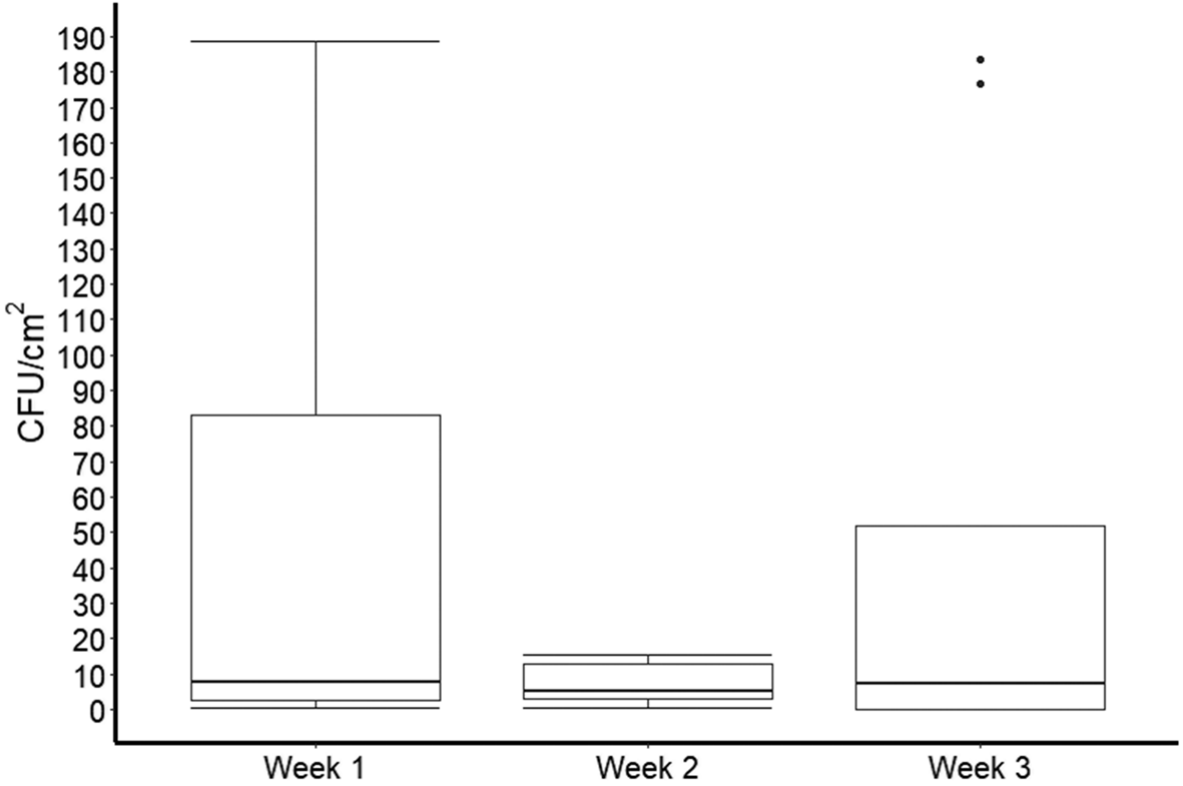
Median with interquartile ranges of aerobic colony forming units (CFU) per cm^2^ of nine sites. This boxplot does not show one extreme outlier (716.67 CFU/cm^2^) from week 2 data

Based on the above results, nine sites were selected for repeated sampling (Table 2). Figure 1 shows that there was no significant change in mean levels of ATP readings over a period of three weeks [*P* = 0.592]. Moreover, as can be seen in Fig. 2, the median CFU per cm^2^ remained largely unchanged over the 3-week period also [*P* = 0.972].

A total of 23 *Escherichia coli* and 6 *Enterococcus* species were recovered during the 3-week study with more than one isolate obtained from some sites. Seven of the nine sites were positive for faecal organisms on more than one occasion.

### Hand samples

*Escherichia coli* were identified in 4 of 51 hand swabs. Of 17 hands sampled in the large animal area, we recovered one *E. coli* isolate from a nurse (resistant to cephalothin and amoxycillin-clavulanate). We sampled 34 hands of veterinary personnel working in the small animal area and found 3 *E. coli* isolates (including 1 AmpC positive-*E. coli*). Faecal bacteria were not identified on the hands of any of 8 students sampled.

### Antimicrobial susceptibility test results

Isolates recovered during a cross-sectional and 3-week longitudinal study in a veterinary hospital that had antimicrobial resistance profiles in common are shown in Table 4.

**Table 4:** Isolates recovered during a cross-sectional and 3-week longitudinal study in a veterinary hospital that had antimicrobial resistance profiles in common. More than one isolate was recovered from some sites; as per protocol, up to 5 isolates of E. coli and enterococci were analysed per sample

**Microorganism**	**Resistance pattern **	**Site **	**Cross-sectional study**	**Longitudinal study **		
				**Week 1**	**Week 2 **	**Week 3**
*Enterococcus faecalis*	TE	Emergency clinic (Table)	+			
		ICU (Floor)				+
	TE, ENR	Dog surgery ward (Shoreline Concrete-Kennel)	+			
		Dog surgery ward (Floor)				+
	Susceptible to all 4 disks tested	Induction room (Anaesthetic machine)	+			
		Consult room (Floor)	+			
		ICU (Fluid pump)	+			
		ICU (Keyboard & mouse)	+			
		ICU (Floor)				+
*Enterococcus faecium*	TE, AMC	Small animal treatment room (Floor)	+			
		Consult room (Keyboard & mouse)	+			
		Dog surgery ward (Door)	+			
		Dog medicine ward (Floor)	+			
	TE, ENR, VA, AMC	Consult room (Couch)	+			
		Small animal treatment room (Telephone)	+			
		Small animal surgery room (Telephone)	+			
		Outside small animal ward (Keyboard & mouse)	+			
		Dog surgery ward (Floor)	+			
	Susceptible to all 4 disks	Dog medicine ward (Kennel door)	+			
		Consult room (Keyboard & mouse)	+			
*Escherichia coli*	TE	Hands of nurse – Large animal area	+			
		Large animal treatment room (Floor)	+			
		ICU (Floor)			+	
	TE, SXT	Large animal treatment room (Keyboard)	+			
		Large animal treatment room (Floor)			+	
		Dog surgery ward (Floor)				+
		Small animal treatment room (Floor)				+
	KF, AMC	Hands of animal care assistant - Dog surgery ward	+			
		ICU (Telephone)	+			
	KF, AMC, CPD	ICU (Floor)	+			
		Dog medicine ward (Floor)	+			
	TE, AMC, SXT	Large animal treatment room (Floor)				+
		Small animal treatment room (Floor)				+
		Dog surgery ward (Floor)				+
	KF, TE, AMC, SXT	Large animal treatment room (Floor)	+			
		Corridor				+
	KF, TE, ENR, AMC, SXT, CPD	Dog surgery ward	+			
		Dog medicine ward (Floor)		+		
	Susceptible to all six disks tested	Induction room (Floor)	+			
		Student computer	+			
		Small animal treatment room (Syringe driver)		+		
		ICU (Floor)		+		
		Dog surgery ward (Floor)		+		
		Large animal treatment room (Floor)			+	
		Large animal treatment room (Keyboard & mouse)				+
		Dog medicine ward (Floor)				+
		Corridor (Floor)				+

*Abbreviations*: *KF *Cephalothin, *TE *Tetracycline, *ENR *Enrofloxacin, *AMC *Amoxycillin-clavulanate, *SXT *Trimethoprim-sulphamethoxazole, *CPD* Cefpodoxime, *VA* Vancomycin

*E. coli* recovered from small animal areas were resistant to cephalothin (9/20), tetracycline (9/20), amoxycillin-clavulanate (9/20), trimethoprim-sulphamethoxazole (8/20), cefpodoxime (6/20), and enrofloxacin (3/20). Isolates of *Enterococcus* species were resistant to tetracycline (17/30), amoxycillin-clavulanate (14/30), enrofloxacin (11/30), and vancomycin (4/30).

In large animal sites, *E. coli* was resistant to tetracycline (9/12), trimethoprim-sulphamethoxazole (7/12), amoxycillin-clavulanate (6/12), cephalothin (4/12), and enrofloxacin (4/12). The number of enterococci identified in large animal sites was less than 10.

**Fig. 3:**
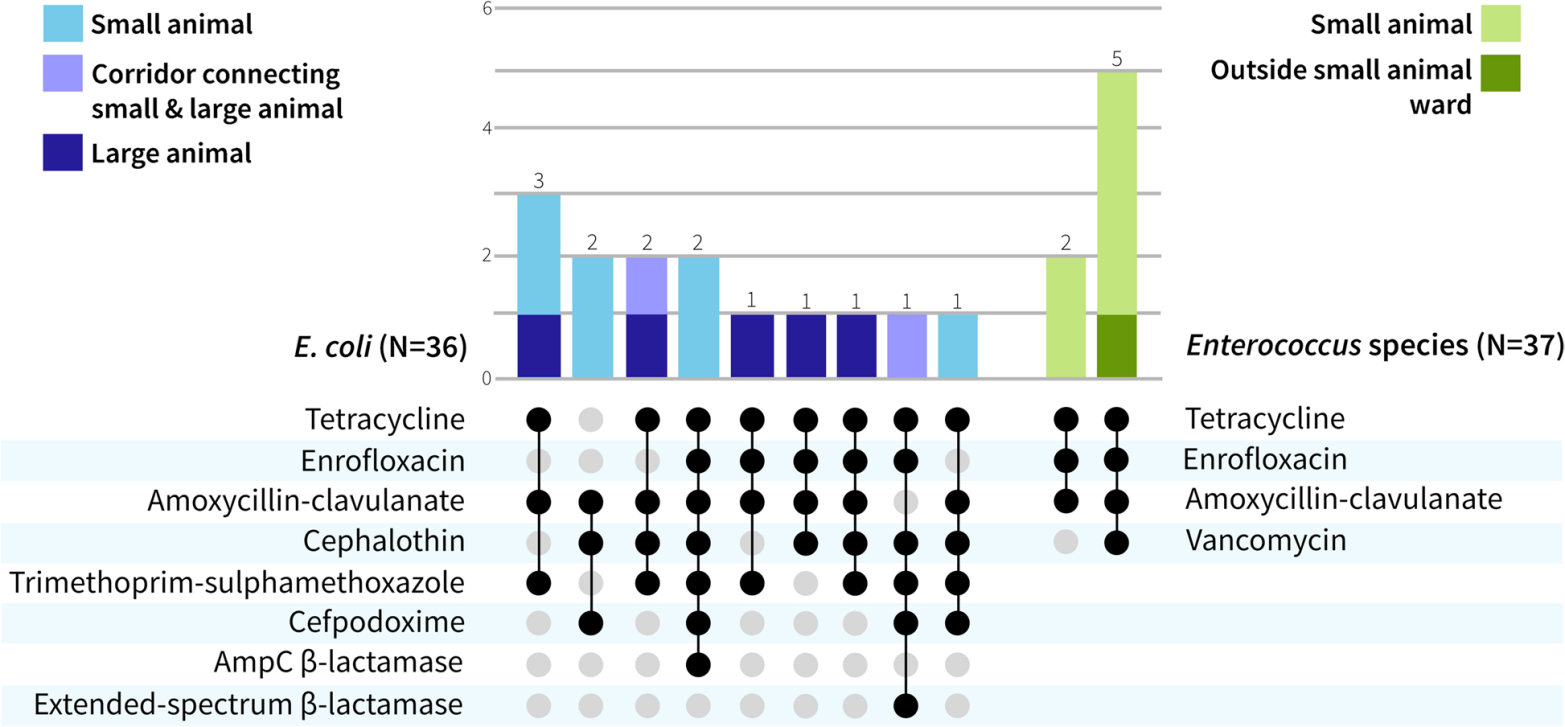
Observed antimicrobial resistance combinations for MDR* E. coli* (left) and MDR *Enterococcus* species (right) and the number of isolates with these combinations (top). MDR is defined as resistance to at least 3 classes of antimicrobials. More than one isolate was recovered from some sites

Of the 36 *E. coli* isolates recovered from the environment during the cross-sectional and longitudinal studies, 15 (41.6%) were resistant to at least 3 different antimicrobial classes (Fig. 3) with no major differences between the percentage of MDR samples identified in small and large animal sites. Seven of 37 (18.9%) enterococci were resistant to at least 3 different antimicrobial classes (Fig. 3). One MDR *Enterococcus* sp. was identified on a keyboard and mouse outside the small animal ward. An ESBL-producing *E. coli* was identified on the corridor connecting small and large animal hospital areas. Two AmpC beta-lactamase-producing *E. coli* were recovered from the dog medicine ward (floor) and the hand of a member of staff in the dog surgery ward. These two AmpC- producing *E. coli* isolates did not have the same resistance profile. Overall, 21 of 73 (28.8%) isolates of faecal bacteria were MDR.

**Fig. 4:**
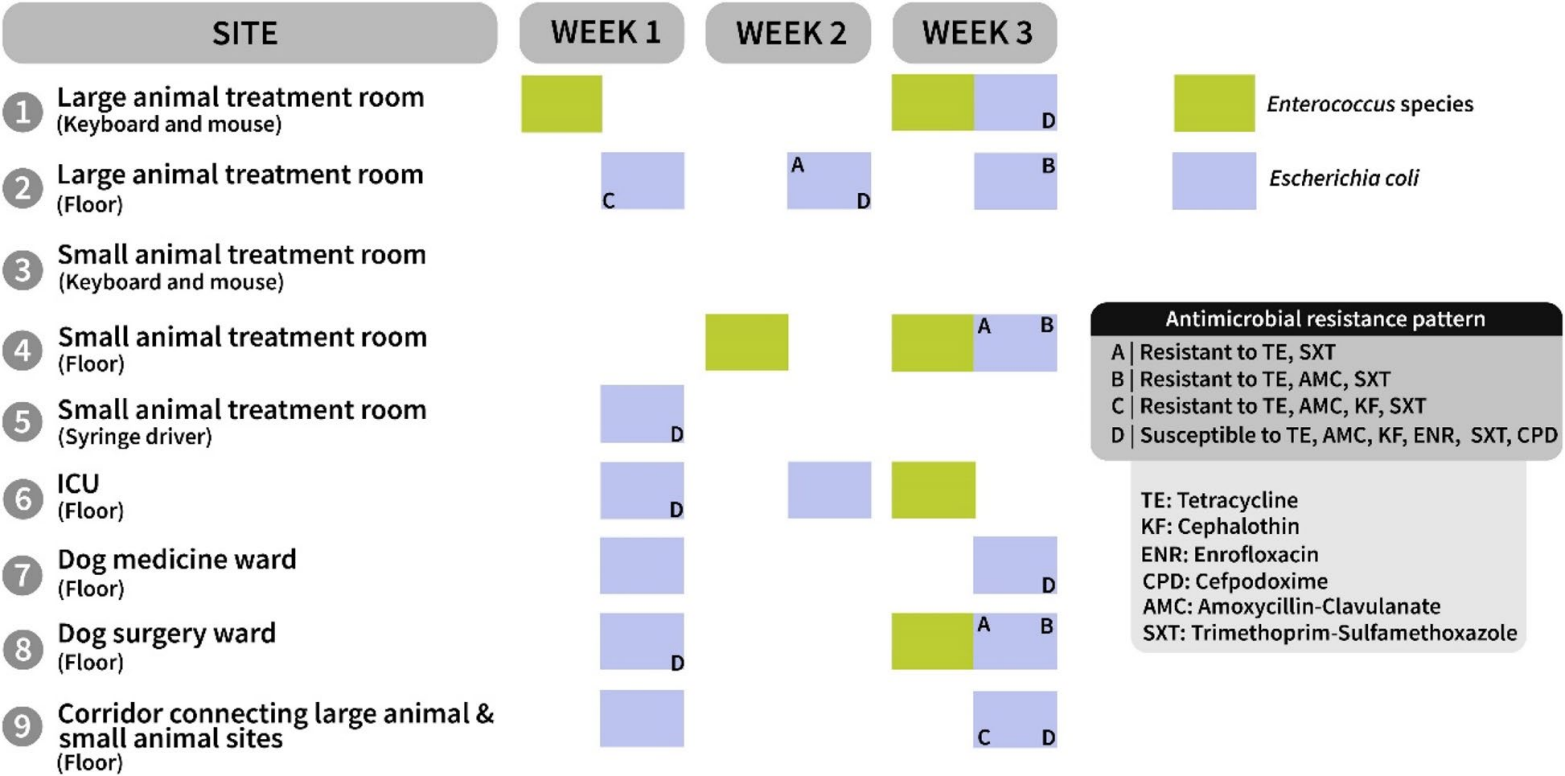
Isolates of* Enterococcus *species (in green) and* E. coli* (in lavender-blue) with the same antimicrobial resistance pattern (A-D) recovered from different sites during a 3-week longitudinal study

Figure 4 shows isolates with the same antimicrobial susceptibility patterns A-D recovered from the 9 sites sampled during the 3-week period of the longitudinal study. *E. coli* isolates with pattern D were isolated in multiple areas in the small animal hospital on week 1, on the floor in the large animal treatment room in week 2 and on the keyboard and mouse in the large animal treatment room, the floor of a kennel in the dog medicine ward and on the corridor between the large and small animal hospitals in week 3.

Isolates of *E. coli* with similar antimicrobial resistant patterns were observed in both the large animal treatment area and the small animal treatment room, and in the large animal treatment room and the corridor connecting the large and small animal hospitals (Table 4. and Fig. 4).

### Retrospective review of patient records

A comparison of the antimicrobial resistance patterns of faecal isolates from the environment and isolates recovered from hospitalised animals during the period of the hygiene studies was conducted to identify possible instances of cross-contamination and the results are presented in Table 5. Some instances of possible cross-contamination and presumptive hospital-acquired infections were detected.

**Table 5:** Faecal bacteria with similar antimicrobial resistance patterns identified in environmental samples and recovered from in-patients during the study period

**Organism**	Resistance pattern^*^	**Site of isolation**	**Date of reporting**
*Escherichia coli*	KF	Dog surgery ward – Floor **Clinical isolate H1—Canine abdominal fluid** Clinical isolate H2—Canine pyometra swab^a^ Clinical isolate H3—Canine urine^a^ Clinical isolate H4—Canine bile^a^	01 June 2021 26 May 2021 26 May 2021 03 June 2021 10 June 2021
	KF, AMC, CPD	ICU – Floor Dog medicine ward – Floor Clinical isolate H5- Canine pleural fluid	26 May 2021 01 June 2021 03 June 2021
	KF, TE, ENR, AMC, SXT	Large animal treatment room – Floor Clinical isolate H6—Canine colon biopsy Clinical isolate H7—Equine nasopharyngeal swab	14 June 2021 28 May 2021 10 June 2021
*Enterococcus faecalis*	TE	Emergency clinic – Table ICU floor Clinical isolate H8—Canine urine	27 May 2021 14 June 2021 17 June 2021
	Resistance not detected to the four AMs tested	Consult room – Floor ICU – Keyboard & mouse ICU – Fluid pump Induction room – Anaesthetic machine **Clinical isolate H9—Canine urine** **Clinical isolate H10—Canine abdominal fluid**^**b**^ Clinical isolate H11—Canine traumatic wound^**b**^	26 May 2021 27 May 2021 11 June 2021 14 June 2021 26 May 2021 26 May 2021 30 June 2021
*Enterococcus faecium*	ENR, AMC, TE	Consult room – Couch^c^ Small animal treatment room – Telephone^c^ Small animal surgery room – Telephone^c^ Outside small animal ward – Keyboard & mouse^c^ Dog surgery ward – Floor^c^ Clinical isolate H12—Canine incision site	26 May 2021 31 May 2021 31 May 2021 31 May 2021 1 June 2021 28 June 2021

Clinical isolates H1, H9 and H10 (in bold) were recovered from the same patient

^*^Antimicrobial resistance patterns of environmental isolates were determined using disc diffusion and of animal isolates by VITEK®2

^a,b^same VITEK resistance pattern

^c^Resistant to vancomycin 30 μg

## Discussion

Maintenance of high standards of hygiene and infection control remains a constant challenge in hospital settings, including veterinary hospitals. Routine hygiene monitoring, including annual hospital-wide checks of bacterial contamination levels in the UCDVH highlighted that some areas were frequently contaminated with faecal organisms. Enterococci and coliforms, including *E. coli*, were the most frequent faecal isolates. In contrast to human hospitals, contamination of surfaces such as floors is a concern in a veterinary setting as, for example, wound dressing of large dogs may be carried out on the floor [18]. Hospital-acquired infections result in a financial burden to both owners and the hospital and in addition, veterinarians who take care of the animals are at risk of acquiring infection.

### Cross-sectional study

The purpose of this study was to investigate in detail possible sources of persistent or repeated contamination to inform necessary changes to infection control protocols. Sites sampled in the cross-sectional study included areas not monitored in previous surveillance sampling, such as corridors, the dispensary and the reception area. Although the results in general indicated satisfactory hygiene levels, with median and interquartile ranges of many samples yielding results below the cut-off values for RLU and CFU/cm^2^, there were several sites where a proportion of samples surpassed the acceptable threshold for CFU/cm^2^ and were contaminated with faecal organisms, including hand touch surfaces such as telephones, keyboards, and mouses. The correlation between RLU and CFU/cm^2^, although statistically significant, was only moderate and faecal organisms were sometimes detected on surfaces that gave results below the cut-off values and thus would have been deemed acceptably clean (Table 3). This is consistent with findings in other studies [19–21] and highlights the fact that while luminometers and other monitoring devices can make a useful contribution to maintaining hygiene levels, they are probably best employed as part of a multimodal approach to improving infection control practices [22].

### Hand hygiene

The detection of faecal contaminants on surfaces such as telephones, keyboards, and mouses as well as on hands confirms that ensuring compliance with hand hygiene protocols can be difficult [23]. Non-compliance with hand hygiene protocols was unexpected as this study took place during the COVID 19 pandemic when hand hygiene was constantly emphasised in all settings. In addition to frequent communications from the UCD Veterinary School Covid-19 Committee on the importance of hand hygiene and other control measures, national advertising campaigns were also in place and thus staff and students were continually reminded of the necessity for compliance with infection control protocols. However, other researchers have also reported slippage in compliance after initial improvements during the Covid-19 pandemic [24].

### Longitudinal study

In the nine sites repeatedly screened during the longitudinal study, the level of bioburden did not fluctuate significantly during the 3-week period. Although variation in interquartile ranges of microbial contamination was observed, similar mean ATP readings and median microbial burdens suggest that cleaning and disinfection practices were consistent and regular. However, results also confirmed the circulation of faecal bacteria within the hospital. Figure 4 shows that in the nine suspected ‘high-risk’ sites screened during the longitudinal study, faecal organisms were repeatedly isolated from some sites. An *E. coli* isolate with antimicrobial-resistant pattern C was first identified on the floor of the large animal treatment room in week 1 and on the corridor in week 3. In week 2, an *E. coli* isolate with pattern A was recovered from the floor of the large animal treatment room and, a week later, from the floor of the dog surgery ward and small animal treatment room. In the third week of the longitudinal study, *E. coli* with pattern B was identified on the floor of the large animal treatment room, small animal treatment room and the dog surgery ward. These data suggest that cross-contamination occurred between different sites in the UCDVH including between large and small animal areas. Contamination of both floors and hand touch surfaces with *E. coli* isolates with resistance pattern D (susceptible to all six antimicrobials tested) in large animal, small animal hospitals, and the corridor connecting them suggests that contamination was likely carried on staff hands and footwear. Previous studies have also reported that veterinarians can disseminate pathogens causing HAI within a small animal hospital [25]. The evidence of cross-contamination was presented to the UCDVH Infection Control Committee and options for limiting movement between different areas of the hospital were discussed. Because of the proximity of the small and large animal hospitals and issues of access to laboratories and other areas, full segregation of staff and students between areas, although ideal, was not deemed feasible or practical. Instead, an alternative route was designated for large animal personnel to access the main hospital building. This route bypasses the main small animal hospital corridors and was deemed preferable for infection control purposes although it is perceived as less convenient by staff. Compliance with using this route is being monitored. Another change in infection control practice was instituted in the form of a monthly hygiene audit of the hospital by the head nurse. This audit, adapted from that available at https://www.thebellamossfoundation.com/hygiene-self-audit, helps to identify any areas where cleaning protocols may require modification or better implementation.

### Retrospective review of patient records

Animals admitted to a veterinary hospital may acquire or shed nosocomial pathogens during their treatment, as indicated in Table 5. In this table, three suspect hospital-acquired infections with *E. coli* (clinical isolates H2-H4) reported in a span of 2 weeks had the same resistance pattern. During the same period, a pure culture of *E. coli* with a similar resistance pattern was isolated from the floor of the dog surgery ward. Examination of the dates of issue of the laboratory reports suggests that environmental contamination may have resulted from the dog infected with isolate H2 which then acted as a source of infection for cases infected with isolates H3 and H4. However, the direction of transmission cannot be determined. Isolates H1, H9 and H10 originated from the same patient. *E. coli* and *Enterococcus faecalis* were identified from the abdominal fluid and urine samples from this patient. *E. faecalis* isolated from the abdominal fluid had the same VITEK resistance pattern as an *E. faecalis* isolated from a traumatic wound in a different patient a few days later. These findings are suggestive of HAI although an alternative explanation could be environmental contamination of samples during collection if aseptic procedures were not strictly followed.

## Limitations

There were some limitations to this study. The number of sites selected for longitudinal sampling was small and the duration of the study was short with sampling conducted over only three weeks. In addition, samples were collected mid-morning to facilitate work practices rather than immediately after cleaning. Collection and analysis of samples immediately after cleaning as well as later in the day would have provided more information on the level and speed at which contamination occurred. In addition, the small number of antimicrobial agents used in susceptibility testing of the environmental isolates and the lack of full genomic typing of isolates was a major limitation. The inability to fully characterise isolates meant that definitive evidence of cross contamination and HAIs could not be generated. Nevertheless, the methods used in this study are those routinely available to veterinary hospitals. The cost of full molecular typing of isolates for infection control purposes, although the method of choice, is likely to be too expensive for most establishments. Limited AR typing as conducted in this study is affordable and, in conjunction with data such as dates and times of contamination/infection, can be valuable in identifying presumptive cross contamination and so prompt a review of infection control procedures to address likely deficiencies.

In conclusion, the results of this study suggest that movement between the small and large animal hospital areas may have been responsible for cross-contamination and possible hospital-acquired infections. The data show that cross-sectional and longitudinal monitoring of faecal contamination across all hospital areas can play an important role in informing review of infection control protocols in veterinary hospital settings.

## Supplementary Information

**Additional file 1:**

## Data Availability

The datasets used and/or analysed during the current study are available from the corresponding author on reasonable request.
