# Supplementary material for: Improving infection control in a veterinary hospital: a detailed study on patterns of faecal contamination to inform changes in practice
Source: Ir Vet J. 2023 Feb 13;76:4. doi: 10.1186/s13620-023-00229-w (PMC9924846; doi:10.1186/s13620-023-00229-w)
Supplement: Supplementary file 1 — Additional file 1: [file 13620_2023_229_MOESM1_ESM.docx]

**Supplementary Data**

| 1. | Site and CFU/cm^2^ |
| --- | --- |
|  | 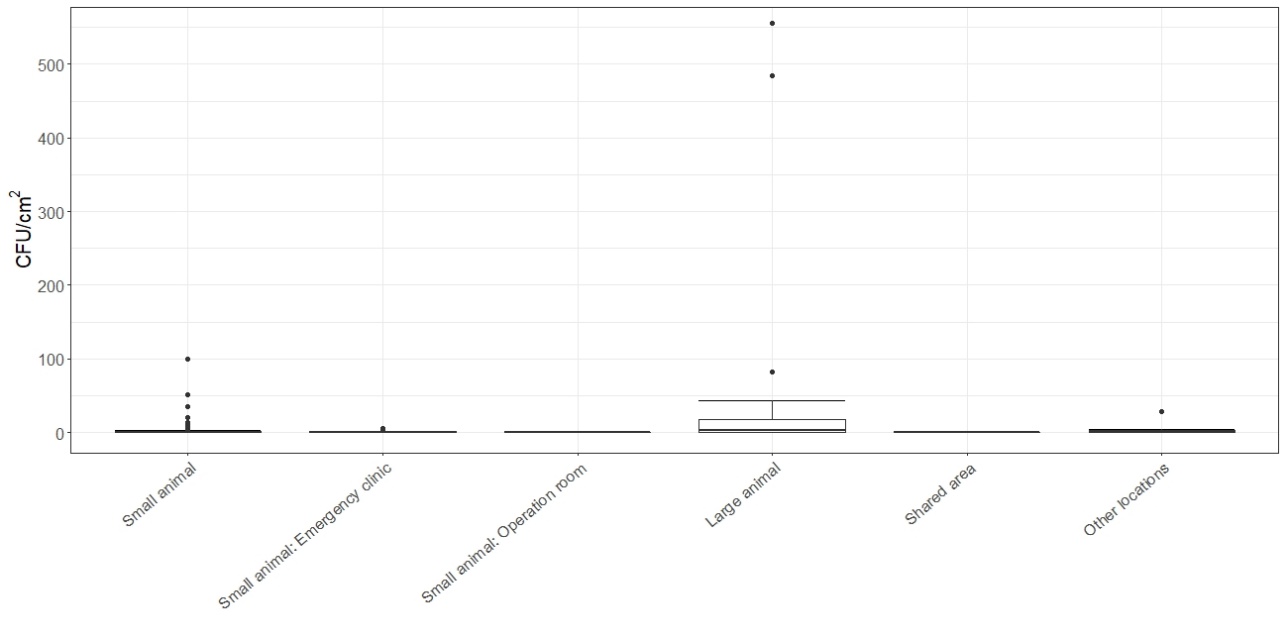  Figure S1. Multiple comparisons of sites and microbial burden (CFU/cm^2^) |
|  | Kruskal-Wallis chi-squared = 14.099, df= 5, p-value = 0.01499  Epsilon squared = 0.126 (small effect size)  No significant difference was found between CFU/cm^2^ and at least one pair of sites when p-values were adjusted with the Holm method. |
|  | |
| 2. | Site and RLU/cm^2^ |
|  | 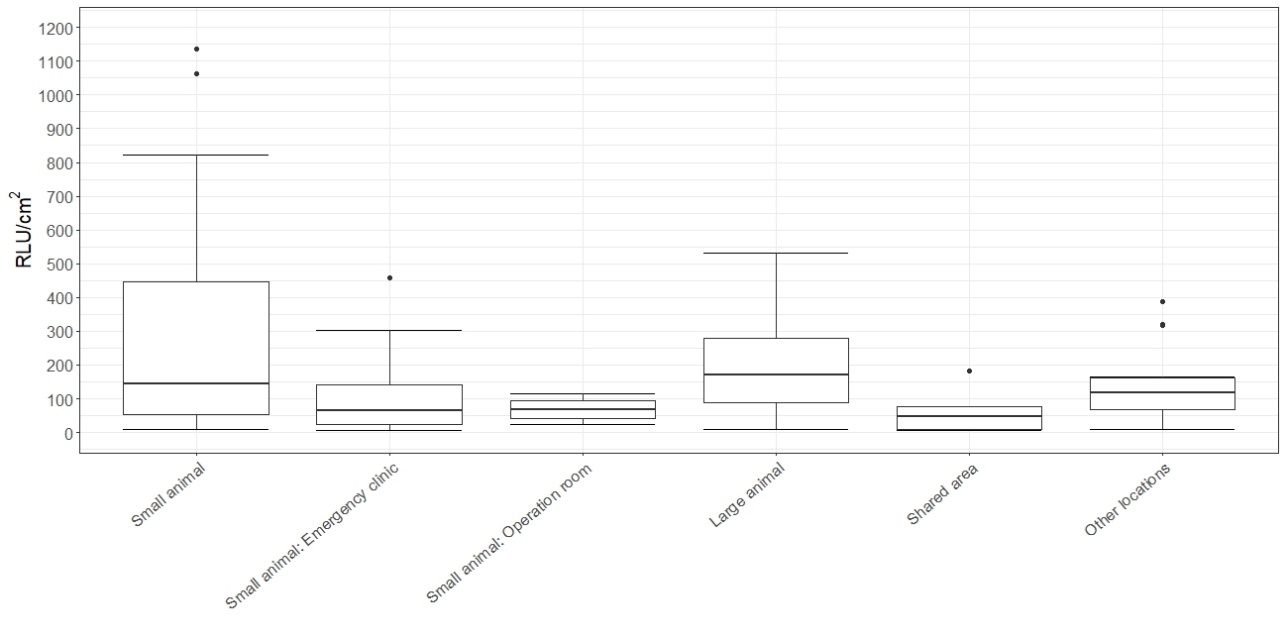  Figure S2. Multiple comparisons of sites and bioburden (ATP readings). |
|  | Kruskal-Wallis chi-squared test = 10.993, df = 5, p-value = 0.05152  Epsilon squared = 0.0982 (negligible effect size)  No significant difference was found between RLU/cm^2^ levels and at least one group of sites. |
| Multiple comparisons of types of surfaces and microbial burden | |
| 3. | Surface and CFU/cm^2^ |
|  | 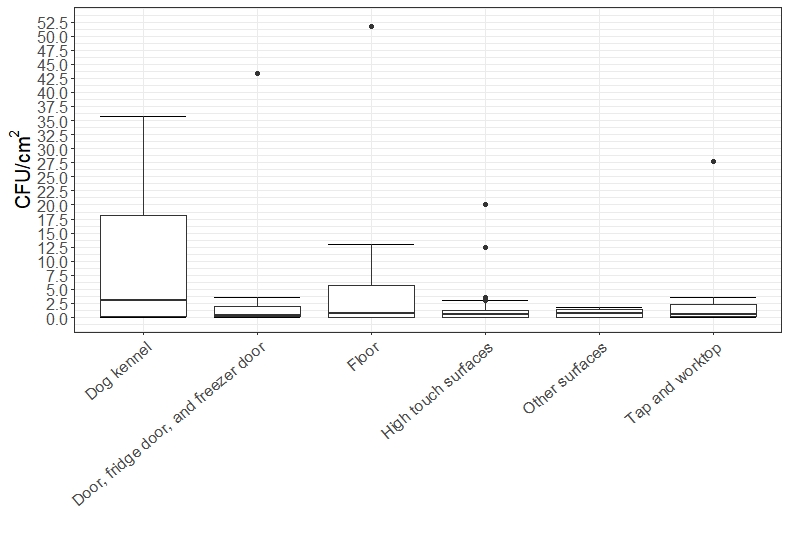  Figure S3. Multiple comparisons of types of surfaces and microbial burden (CFU/cm^2^).  Following outliers not shown tap and worktop – 555.5556 CFU/cm^2^, dog kennel – 100 CFU/cm^2^, high touch surfaces – 484.44 CFU/cm^2^, 82.5 CFU/cm^2^. |
|  | Kruskal-wallis chi-squared = 2.7938, df = 5, p-value = 0.7317  Epsilon squared = 0.0249  Significant difference was not found between CFU/cm^2^ levels and at least one group of surfaces. |
| Multiple comparisons of types of surfaces and bioburden | |
| 4. | Surface and RLU/cm^2^ |
|  | 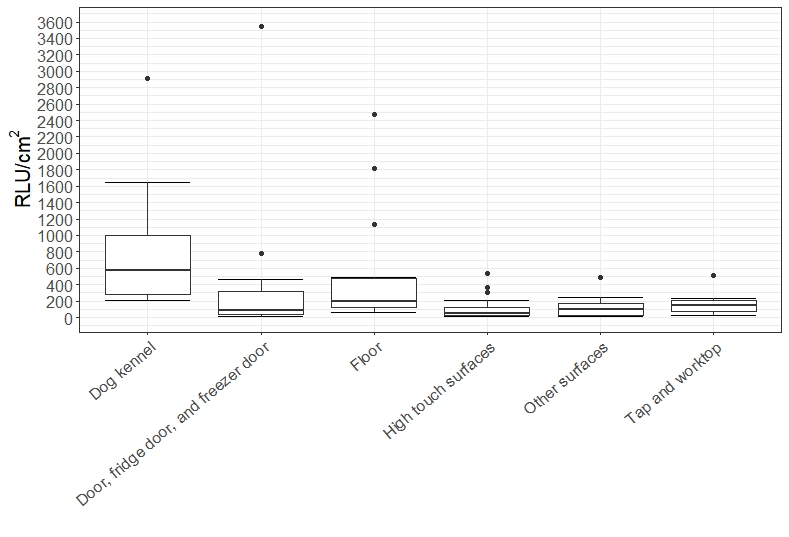  Figure S4. Multiple comparisons of types of surfaces and bioburden (ATP readings). One outlier 5996.497 RLU/cm2 from door, fridge, and freezer door not shown. |
|  | Kruskal-Wallis chi-squared = 35.457, df = 5, p-value = 1.22e-06  Epsilon squared = 0.317  Significant difference was found between RLU/cm^2^ levels and at least one group of sites.  List of pair of surfaces which were significantly different   1. Dog kennel – Door, fridge door, and freezer door (p=0.0125) 2. Dog kennel – High touch surface (p = 8.66 x 10^-6^) 3. Floor – High touch surface (HTS) (p=3.56 x 10^-4^) 4. Dog kennel – Other (p=0.0267) |
